# Association of Maternal Preeclampsia with Neonatal Respiratory Distress Syndrome in Very-Low-Birth-Weight Infants

**DOI:** 10.1038/s41598-019-49561-8

**Published:** 2019-09-13

**Authors:** Yu-Hua Wen, Hwai-I. Yang, Hung-Chieh Chou, Chien-Yi Chen, Wu-Shiun Hsieh, Kuo-Inn Tsou, Po-Nien Tsao, Taiwan Premature Infant Developmental Collaborative Study Group

**Affiliations:** 1 Department of Pediatrics, National Taiwan University Hospital, National Taiwan University College of Medicine, Taipei, Taiwan; 2 Genomics Research Center, Academia Sinica, Taipei, Taiwan; 3 Institute of Clinical Medicine, National Yang-Ming University, Taipei, Taiwan; 4 Department of Pediatrics, Cathay General Hospital, Taipei, Taiwan; 5 Premature Baby Foundation of Taiwan, Taipei, Taiwan; 6 Department of Pediatrics, Cardinal Tien Hospital and College of Medicine, Fu Jen Catholic University, New Taipei City, Taiwan; 7 The Research Center for Developmental Biology and Regenerative Medicine, National Taiwan University, Taipei, Taiwan

**Keywords:** Neonatology, Preterm birth, Preterm birth, Respiratory distress syndrome, Paediatric research

## Abstract

Preeclampsia is a common cause of preterm birth and neonatal morbidity, but its relationship with neonatal respiratory distress syndrome (RDS) remains controversial. We conducted a retrospective cohort study with data from very-low-birth-weight (VLBW) infants born in 1997–2014 from the database of the Premature Baby Foundation of Taiwan to evaluate the relationship between maternal preeclampsia and neonatal RDS. In total, 13,490 VLBW infants were enrolled, including 2200 (16.3%) infants born to preeclamptic mothers. The mean (standard deviation) gestational ages were 30.7 (2.5) weeks in the preeclamptic group and 28.6 (2.9) weeks in the control (non-preeclamptic) group. Severe RDS was defined according to the surfactant therapy requirement. The incidence of severe RDS was lower in infants exposed to maternal preeclampsia than in controls [28.9% vs. 44%; odds ratio (OR), 0.52; 95% confidence interval (CI), 0.47–0.57]. However, after adjustment for confounders, the OR for severe RDS development in the preeclampsia group was 1.16 (95% CI, 1.02–1.31). Other factors, such as gestational age, birth weight, female sex, and antenatal receipt of two or more steroid doses were significantly protective against RDS in multivariate regression analysis. This study revealed that maternal preeclampsia slightly increases the risk of severe RDS in VLBW infants.

## Introduction

Preeclampsia is a systemic syndrome that occurs in 5–10% of pregnant women and is a leading cause of maternal and neonatal morbidity and mortality^1^. This syndrome poses a great challenge for obstetricians and neonatologists because the optimal timing for delivery is a dilemma. In most cases, preeclampsia occurs at the late preterm or term stage, but about 12% of affected women develop early-onset preeclampsia (at <34 weeks of gestation)^2^. The etiology of early-onset preeclampsia differs from that of late-onset preeclampsia; abnormal placentation and spiral artery remodeling lead to intrauterine growth restriction and a greater probability of preterm birth^3–5^. The rates of perinatal death and severe neonatal morbidity are much higher in association with early-onset relative to late-onset preeclampsia^2^.

Respiratory distress syndrome (RDS) is among the most common complications of preterm delivery. Preterm RDS is secondary to surfactant insufficiency, and its incidence is usually related inversely to gestational age (GA). The literature contains conflicting information on the effect of preeclampsia per se on RDS^6–18^. Shah *et al*.^12^ found a lower incidence of RDS in preterm (<34 gestational weeks) infants born to preeclamptic mother than in a control group. In contrast, Tagliaferro *et al*.^18^ found in a cohort study that the risk of severe RDS was increased in extremely premature (23–28 gestational weeks) infants exposed to preeclampsia. Thus, this study aimed to analyze the relationship between maternal preeclampsia and the development of RDS in very-low-birth-weight (VLBW) infants in a large cohort.

## Results

### Baseline characteristics of study participants

In total, 13,490 VLBW infants, including 2,200 (16.3%) cases born to mothers with preeclampsia, were enrolled in this study. The mean (standard deviation) GAs were 30.7 (2.5) weeks in the preeclamptic group and 28.6 (2.9) weeks in the control group (odds ratio (OR), 1.30; 95% confidence interval (CI), 1.28–1.32; *P* < 0.001; Table 1). The overall incidence of RDS was about 86%, and that of severe RDS (requiring surfactant use) was 41.2%. The baseline characteristic that differed most between the preeclampsia and control groups was the proportion of small-for-gestational-age (SGA) infants (72.4% vs. 24.9%; OR, 7.91; 95% CI, 7.14–8.77; Table 1). The GA, birth weight, female predominance, and Cesarean section rate were also significantly greater in the preeclamptic group than in the control group (Table 1). The incidence of RDS in infants was lower in the presence than in the absence of preeclampsia (80.9% vs. 87.2%; OR, 0.58; 95% CI, 0.52–0.66; Table 1). The incidence of surfactant use was also significantly lower in the preeclampsia group than in the control group (28.9% vs. 44%; OR, 0.52; 95% CI, 0.47–0.57; Table 1).

**Table 1:** Demographic and clinical variables for VLBW infants born to mothers with and without preeclampsia.

Variable	Preeclampsia (N = 13490)			
	No (N = 11290)	Yes (N = 2200)	OR	*p* value
**Gender, N (%)** ^1^				
Female	5381 (47.7)	1170 (53.3)	Reference	
Male	5896 (52.3)	1026 (46.7)	0.80 (0.73–0.88)	<0.001
GA, Mean(SD)	28.6 (2.9)	30.7 (2.5)	1.30 (1.28–1.32)	<0.001
BBW, Mean(SD) /100g^2^	11.2 (2.7)	11.3 (2.6)	1.02 (1–1.04)	0.032
**Small gestational age, N (%)** ^3^				
No	8428 (75.1)	606 (27.6)	Reference	
Yes	2798 (24.9)	1592 (72.4)	7.91 (7.14–8.77)	<0.001
**Number of antenatal steroid using, N (%)** ^4^				
<1	6281 (58.9)	1195 (56.8)	Reference	
≥2	4376 (41.1)	909 (43.2)	1.09 (0.99–1.2)	0.069
**RDS, N (%)** ^5^				
No	1440 (12.8)	440 (20.0)	Reference	
Yes	9839 (87.2)	1756 (80.0)	0.58 (0.52–0.66)	<0.001
**Surfactant, N (%)** ^6^				
No	6286 (56.0)	1551 (71.2)	Reference	
Yes	4930 (44.0)	629 (28.9)	0.52 (0.47–0.57)	<0.001
**Cesarean section, N (%)** ^7^				
No	4467 (39.7)	166 (7.6)	Reference	
Yes	6788 (60.3)	2031 (92.4)	8.05 (6.84–9.47)	<0.001
**Singleton, N (%)** ^8^				
No	3560 (31.6)	346 (15.8)	Reference	
Yes	7690 (68.4)	1847 (84.2)	2.47 (2.19–2.79)	<0.001
Days on IPPV, Mean(SD)^14^	13.2 (24.4)	6.8 (16.5)	0.98 (0.98–0.98)	<0.001
Days on CPAP or IPPV, Mean(SD)^15^	41.5 (39.3)	27.7 (31.8)	0.99 (0.99–0.99)	<0.001

Abbreviations: VLBW, very low birth weight. GA, gestational age. BBW, birth body weight. RDS, respiratory distress syndrome. IPPV, intermittent positive pressure ventilation. CPAP, continuous positive airway pressure

### Findings of multivariate analysis

In a multivariate logistic regression analysis including GA, birth weight, infant sex, SGA, and antenatal steroid use as potential confounders, preeclampsia was associated with an insignificantly increased risk of RDS (adjusted odds ratio (aOR), 1.12; 95% CI, 0.98–1.29; Table 2), but a significantly increased risk of severe RDS development (aOR, 1.16; 95%CI, 1.02–1.31; Table 3). GA, but not birth weight, was associated negatively with RDS. GA and birth weight were associated inversely with severe RDS, with aOR of 0.68 (95% CI, 0.65–0.7) and 0.94 (95% CI, 0.91–0.97), respectively. Male infants were more likely than females to develop RDS and severe RDS [aOR (95%CI), 1.18 (1.06–1.32) and 1.24 (1.14–1.35), respectively]. SGA and antenatal use of two or more doses of steroids protected against the development of severe RDS [aOR (95% CI), 0.8 (0.69–0.93) and 0.57 (0.53–0.62), respectively].

**Table 2:** Odds of RDS development according to various factors.

Variable	RDS (yes vs. no)			
	Univariate model		Model 1	
	Odds ratio (95%CI)	*p* value	Adjusted odds ratio (95%CI)	*p* value
GA	0.69 (0.68–0.71)	<0.0001	0.71 (0.68–0.74)	<0.0001
BBW (per 100 grams)	0.78 (0.76–0.79)	<0.0001	0.97 (0.93–1.01)	0.123
Gender (male vs. female)	1.30 (1.18–1.43)	<0.0001	1.18 (1.06–1.32)	0.003
SGA (yes vs. no)	0.28 (0.25–0.31)	<0.0001	0.8 (0.67–0.97)	0.022
Antenatal steroid using (≥2 vs.<2)	1.00 (0.90–1.11)	0.983	0.79 (0.71–0.88)	<0.0001
Preeclampsia (yes vs. no)	0.58 (0.52–0.66)	<0.0001	1.12 (0.98–1.29)	0.095

Abbreviations: RDS, respiratory distress syndrome. GA, gestational age. BBW, birth body weight. SGA, small for gestational age.

**Table 3:** Odds of severe RDS development according to various factors.

Variable	Severe RDS (yes vs. no)			
	Univariate model		Model 1	
	Odds ratio (95%CI)	*p* value	Adjusted odds ratio (95%CI)	*p* value
GA	0.65 (0.66–0.64)	<0.001	0.68 (0.65–0.7)	<0.001
BBW (per 100 grams)	0.73 (0.75–0.72)	<0.001	0.94 (0.91–0.97)	0.0003
Gender (male vs. female)	1.29 (1.38–1.20)	<0.001	1.24 (1.14–1.35)	<0.001
SGA (yes vs. no)	0.33 (0.36–0.30)	<0.001	0.8 (0.69–0.93)	0.005
Antenatal steroid using (≥2 vs.<2)	0.70 (0.75–0.65)	<0.001	0.57 (0.53–0.62)	<0.001
Preeclampsia (yes vs. no)	0.52 (0.57–0.47)	<0.001	1.16 (1.02–1.31)	0.026

Abbreviations: RDS, respiratory distress syndrome. GA, gestational age. BBW, birth body weight. SGA, small for gestational age.

### Findings of subgroup analysis with stratification by SGA

Given the uneven distribution of baseline SGA between the preeclampsia and control groups, and the possible interaction between preeclampsia and SGA in the effect on RDS, we performed a subgroup analysis with stratification by SGA. No significant association between preeclampsia and severe RDS was found in the SGA or non-SGA subgroup (OR (95% CI), 1.12 (0.94–1.34) and 1.15 (0.95–1.40), respectively; Table 4). The trends of other predictor variables were consistent with those revealed by the whole-sample multivariate regression analysis.

**Table 4:** Risk of severe RSD development according to SGA status.

	Sever RDS (yes vs. no)			
	Non-SGA		SGA	
	aOR (95%CI)	*p* value	aOR (95%CI)	*p* value
Preeclampsia (yes vs. no)	1.15 (0.95–1.40)	0.142	1.12 (0.94–1.34)	0.218
GA	0.72 (0.69–0.76)	<0.001	0.62 (0.58–0.66)	<0.001
BBW (per 100 grams)	0.93 (0.89–0.97)	0.001	0.92 (0.87–0.97)	0.004
Gender (male vs. female)	1.27 (1.16–1.40)	<0.001	1.23 (1.03–1.47)	0.024
Antenatal steroid using (≥2 vs.<2)	0.55 (0.50–0.60)	<0.001	0.61 (0.51–0.73)	<0.001

Abbreviations: RDS, respiratory distress syndrome. SGA, small for gestational age. GA, gestational age. BBW, birth body weight.

## Discussion

In this cohort study of VLBW infants, the incidences of RDS and severe RDS were lower in infants whose mothers had preeclampsia; we infer that more protective factors were present in the preeclampsia group than in the control group. This assumption was verified by those of the multivariate analysis, which showed that maternal preeclampsia increased the risk of severe RDS, but not RDS, after adjustment for confounding factors. The high incidence or diagnostic rate of RDS of any grade in very preterm infants in our sample may have obscured a difference in RDS risk between the preeclampsia and control groups. The need for surfactant use, which was generally regarded as rescue therapy in Taiwan during our study period (1997–2014), may more objectively represent the presence and the severity of RDS. GA, birth weight, female sex, and antenatal receipt of two or more doses of steroids were protective factors against RDS in this study.

Our findings are contrary to previous findings that preeclampsia is a protective factor against RDS^7,12^. Those previous studies promoted the belief that maternal preeclampsia accelerates fetal lung maturation. Some trial results also supported the enhancement of biochemical lung maturation profile by chronic in utero stress, possibly via increased fetal cortisol production^8,9^. The most recent study to support such a protective effect of preeclampsia on RDS was a large cohort study conducted in the Netherlands^10^. However, that study was conducted to examine the outcomes of late-onset preeclampsia (34–37 gestational weeks). Our preeclampsia group contained mainly early-onset preeclampsia cases, with a different pathogenesis and worse neonatal outcomes relative to late-onset preeclampsia^2^.

On the other hand, accumulating data indicate the absence of a protective effect of maternal preeclampsia on the fetal respiratory system^6,11,13^. Even more, preeclampsia was associated with an increased risk of neonatal RDS in several studies, consistent with our findings^14–19^. In a large national cohort study conducted in the United States, which included 156,681 infants born to mothers with preeclampsia, the greater probability of RDS was found in the preeclamptic group compared to the non-preeclamptic group (6.6% vs. 1.9%)^17^. Another recent single-center cohort study including infants born at 23–28 gestational weeks also revealed that preeclampsia increased the risk of severe RDS^18^. Preeclampsia is characterized by an imbalanced maternal angiogenic state, resulting in generalized endothelial dysfunction, increased levels of maternal antiangiogenic factor soluble fms-like tyrosine kinase-1 (sFlt-1), and decreased free circulating levels of the angiogenic factors vascular endothelial growth factor (VEGF) and placental growth factor^20–23^. VEGF is important for normal lung vasculature^24^ and surfactant protein production^25–27^. sFlt-1, an antagonist of VEGF, can impede VEGF signaling and lead to impaired surfactant production. RDS is secondary to surfactant insufficiency, and low VEGF concentrations have been associated with RDS severity in preterm infants^28–31^. Similarly, high sFlt-1 and/or low VEGF levels have been observed in neonatal cord blood and tracheal aspirates from infants born to preeclamptic mothers^32–36^. Higher sFlt-1 concentrations have also been noted in amniotic fluid from preeclamptic mother^34^. Wang *et al*.^16^ demonstrated that preeclampsia was correlated with a higher maternal circulating sFlt-1 level and an increased risk of neonatal RDS. All of these findings imply that preeclampsia creates not only a stressful intrauterine environment, but also an unfavorable state for fetal lung surfactant production. A study of infants born prematurely to mothers with severe preeclampsia or amniotic infection found that non-surviving infants exposed to preeclampsia had a higher proportion of accelerated morphologic lung maturation than non-surviving infants exposed to amniotic infection (40% vs. 5%). However, the remaining survivors in the preeclampsia group needed more respiratory support after the first 24 hours, indicating greater surfactant insufficiency^37^.

In the present study, baseline characteristics showed a high degree of heterogeneity between the preeclampsia and control groups. Infants born to preeclamptic mothers had significantly greater birth weights and GAs, and the female predominance and SGA proportion were greater in this group than in the control group. SGA status is a common complication of preeclampsia, with a widely ranging incidence of 13.36–52%^11,14,38–40^. In this study, the incidence of SGA in the preeclamptic group was very high, perhaps due to the application of inclusion criteria according to birth weight instead of GA, and thus the enrollment of more SGA infants. Although the association between preeclampsia and severe RDS was insignificant in our subgroup analysis, the trend of increased risk was consistent in both groups. This finding indicates that preeclampsia and SGA do not interact significantly in affecting RDS. In the whole-sample multiple regression analysis, 95% CIs for the association of preeclampsia with severe RDS were very close to 1, which may explain the failure to detect a significant difference in the subgroup analysis in this small sample.

Birth weight and GA are known to correlate negatively with RDS; male sex also contributes to an increased RDS risk^41^. Amorim *et al*.^42^ reported that antenatal corticosteroid therapy could reduce the risk of RDS in the presence of severe preeclampsia at 26–34 weeks of gestation. Our study revealed a weak positive association between preeclampsia and severe RDS, with other covariates also contributing to neonatal RDS development. Thus, the relationship between preeclampsia and RDS could be divergent if all confounding factors are not taken into consideration. This situation may reasonably explain the discrepant results obtained in previous studies, given the small sample and examination of different potentially confounding factors.

The main strength of our study was the examination of a large cohort of VLBW neonates; the mean gestational age was 30 weeks, which indicated that most mothers with preeclampsia had the early-onset form of this condition. We also excluded mother with histories of chronic hypertension, which may alter the pathogenesis and effects relative to those of maternal preeclampsia alone. Another large cohort study of infants delivered at >23 gestational weeks’ (average 37 weeks’) revealed an increased risk of RDS in the presence of maternal hypertensive disorder, and different neonatal outcomes among cases of maternal gestational hypertension, mild chronic hypertension, and mild preeclampsia^15^.

Our study has some limitations. First, the reliability of our data depends on the accuracy of pediatricians’ records, but the large sample size minimizes the potential effect of this factor. Second, maternal comorbidity, body mass index, and severity of preeclampsia were not recorded in the neonatal-oriented registration database. Third, the type and total dosage of antenatal steroids, and the interval between their use and delivery, were not recorded in the database. Additionally, RDS diagnoses were based on physicians’ subjective interpretations of clinical and chest X-ray data, which may cause bias. For this reason, we took surfactant usage as an objective indicator of severe RDS.

## Conclusions

In this population-based cohort study of VLBW infants, we found early-onset maternal preeclampsia slightly increased the risk of severe RDS compare with preterm delivery of other causes, whereas GA, birth weight, SGA, female sex and antenatal use of two or more doses of steroids were prominent protective factors that decreased the risk of RDS.

## Methods

### Study subjects

This study was based on data on all VLBW infants born from 1997 to 2014 in all 22 neonatal departments in Taiwan, registered in the database of the Premature Baby Foundation of Taiwan. VLBW was defined as birth body weight <1500 g^43^. The data collected included antenatal and perinatal histories, delivery mode, neonatal histories including diagnoses, complications during hospitalization, and clinical outcomes at discharge. Patient information received by the database coordinator was cross checked with the national birth registry. The exclusion criteria were congenital anomalies, chromosomal anomalies, and maternal chronic hypertension with or without preeclampsia. The cases were divided into the preeclampsia and control (non-preeclampsia) groups. Preeclampsia was defined as diastolic blood pressure ≥90 mm Hg with proteinuria ≥1+ (30 mg/dl) on dipstick testing or non-dependent edema during pregnancy^44^.

### Ethical considerations

Written informed consent was obtained from included subjects’ parents or legal guardians. The study was approved by the Institutional Review Boards of eight participating hospitals (National Taiwan University Hospital, Chang Gung Memorial Hospital, China Medical University Hospital, National Cheng Kung University Hospital, Tri-Service General Hospital, Chung Shan Medical University Hospital, Shin Kong Wu Ho-Su Memorial Hospital, and Kaohsiung Medical University Chung-Ho Memorial Hospital), and Joint Institutional Review Board for the other participating hospitals. All research was performed in accordance with relevant guidelines and regulations.

### Outcome variables

RDS was diagnosed by neonatologists in charge of the infants’ care according to clinical symptoms and signs, chest X-ray findings, and arterial blood gas findings. Mainstream surfactant therapy was used as a rescue treatment rather than a prophylactic during the study collection period in Taiwan. Thus, we defined severe RDS according to the requirement for surfactant therapy. Antenatal steroid usage was defined as the receipt of any type of steroid prior to delivery to accelerate fetal lung maturation. SGA status was defined as birth body weight <10th percentile for gestational age^45^.

### Statistical analysis

The chi-squared test and Student’s *t*-test were used to compare the distributions of categorical and continuous variables, respectively, between groups. A multivariate logistic regression model was used to analyze the association between maternal preeclampsia and RDS risk, with adjustment for potential confounders. The confounders included demographic and clinical variables that differed between the preeclampsia and control groups in univariate analysis. aORs with 95% CIs were calculated to assess the magnitudes of the associations between various factors and RDS risk. Significance was determined by two- tailed *P* < 0.05. The association between preeclampsia and RDS was further examined in a subgroup analysis stratified according to SGA.

## Data Availability

The datasets used and/or analyzed during the current study are available from the corresponding author on reasonable request.
